# Editorial Brevities

**Published:** 1886-12-20

**Authors:** 


### EDITORIAL BREVITIES.

WE observe that the article on Duodeno-Cholecystostomy contributed by Dr. J. McF. Gaston, of this city, to the Reference Handbook of the Medical Sciences, has been translated and republished, with the illustrations, in the Journal de Medicine of Paris. It may be noticed, in connection with this recognition of Professor Gaston abroad, that he has recently performed a nephrotomy and removed two urinary calculi from the opening of the ureter, and, on the 25th of November, he ligated the femoral artery below scarpa triangle in a case of popliteal aneurism.

